# Enabling Single‐Cell Drug Response Annotations from Bulk RNA‐Seq Using SCAD

**DOI:** 10.1002/advs.202204113

**Published:** 2023-02-10

**Authors:** Zetian Zheng, Junyi Chen, Xingjian Chen, Lei Huang, Weidun Xie, Qiuzhen Lin, Xiangtao Li, Ka‐Chun Wong

**Affiliations:** ^1^ Department of Computer Science City University of Hong Kong Kowloon Hong Kong; ^2^ The Laboratory of Data Discovery for Health (D²4H), Hong Kong Science Park New Territories Hong Kong; ^3^ College of Computer Science and Software Engineering, Shenzhen University Shenzhen China; ^4^ School of Artificial Intelligence Jilin University Jilin China; ^5^ Shenzhen Research Institute City University of Hong Kong Shenzhen China; ^6^ Hong Kong Institute for Data Science City University of Hong Kong Kowloon Hong Kong

**Keywords:** drug response annotation, single‐cell sequencing, transfer learning

## Abstract

The single‐cell RNA sequencing (scRNA‐seq) quantifies the gene expression of individual cells, while the bulk RNA sequencing (bulk RNA‐seq) characterizes the mixed transcriptome of cells. The inference of drug sensitivities for individual cells can provide new insights to understand the mechanism of anti‐cancer response heterogeneity and drug resistance at the cellular resolution. However, pharmacogenomic information related to their corresponding scRNA‐Seq is often limited. Therefore, a transfer learning model is proposed to infer the drug sensitivities at single‐cell level. This framework learns bulk transcriptome profiles and pharmacogenomics information from population cell lines in a large public dataset and transfers the knowledge to infer drug efficacy of individual cells. The results suggest that it is suitable to learn knowledge from pre‐clinical cell lines to infer pre‐existing cell subpopulations with different drug sensitivities prior to drug exposure. In addition, the model offers a new perspective on drug combinations. It is observed that drug‐resistant subpopulation can be sensitive to other drugs (e.g., a subset of JHU006 is Vorinostat‐resistant while Gefitinib‐sensitive); such finding corroborates the previously reported drug combination (Gefitinib + Vorinostat) strategy in several cancer types. The identified drug sensitivity biomarkers reveal insights into the tumor heterogeneity and treatment at cellular resolution.

## Graphical abstract

A transfer learning framework for single cell drug response prediction. This framework integrates domain adaptation to learn cell line bulk pharmacogenomics and transfers the knowledge to infer drug sensitivities at single cells before treatment. This ranking‐based framework for drug sensitivity inference provides new strategies to account for intratumoral heterogeneity; providing new perspectives for biomarker discovery and drug combination applications.

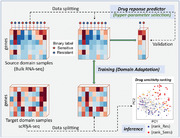

## 1 Introduction

The personalized anti‐cancer therapies have achieved successes in several malignancies.^[^ ^1^ ^]^ However, even with positive responses after treatment, the existence of intratumoral heterogeneity can ultimately drive tumor progression or metastasis as one of the key factors contributing to lethal outcomes.^[^ ^2^ ^]^ The intratumoral heterogeneity is caused by many factors, such as genetic heterogeneity of cells^[^ ^3^ ^]^ and the existence of multiple independent cell subpopulations within a tumor.^[^ ^4^ ^]^ The intratumor heterogeneity is a well‐known issue leading to treatment failure.^[^ ^4^ ^]^ Currently, there are past studies which have evaluated the selective pressure after drug treatment using single cell data; for instance, genomics and single‐cell phosphoprotomics analysis on patient‐derived epidermal growth factor receptor (EGFR)‐mutated in vivo mTOR kinase inhibitor (mTORki) resistance glioblastoma (GBM) model have been proposed. Researchers identified promising drug combinations by resolving the related protein signal networks.^[^ ^5^ ^]^ Moreover, the drug combination efficacy was validated with the complete and lasting tumor suppression on GBM39 implanted mice model.^[^ ^5^ ^]^ Another research uses single‐cell proteomics to study the drug‐induced (anti‐EGFR therapy) resistance and tumor phenotypic switch (a switch from one signaling state to another state) in triple‐negative breast cancer (TNBC).^[^ ^6^ ^]^ EGFR is highly expressed in TNBC and was suggested as a therapeutic target.^[^ ^7^ ^]^ However, anti‐EGFR monotherapies for TNBC are normally failed in clinical.^[^ ^6^ ^]^ Researchers demonstrated that distinct cell subpopulations could independently grow in response to different anti‐cancer treatments. Such kind of anti‐cancer progression may be due to the expansion of previously untargeted (no tailored accurately) or undetected cell subclones.^[^ ^6^ ^]^ In addition, the treatment that does not target estrogen receptor (ER) may result in incomplete suppression of cancer cells and lead to a phenotype switch from TNBC to ER‐positive enrichment.^[^ ^6^ ^]^ In contrast, anti‐EGFR therapy may benefit when combined with anti‐ER treatment; it is suggested by the patient‐specific signaling signature (PaSSS) based predictions and was experimentally validated in two TNBC cell lines.^[^ ^6^ ^]^

However, unlike studies that were currently limited to a few cancer types and a small subset of drugs at the single‐cell level,^[^ ^5^ ^]^ more studies are mainly based on the tumor data profiled in bulk RNA‐seq. Among them, cancer cell lines are still the most widely applied laboratory models for oncology study and anti‐cancer drug discovery.^[^ ^8^ ^]^ In contrast to the limited drug sensitivity information in single cells, there are numerous drug efficacy profiling and studies in cancer cell lines. For example, in the Genomics of Drug Sensitivity in Cancer database (GDSC), over 1000 human cancer cell lines were screened with a variety of anti‐cancer compounds including both cytotoxic chemotherapeutics and targeted agents that were clinically approved or undergoing clinical development,^[^ ^9^ ^]^ allowing the identification of transcriptome‐based (bulk RNA‐seq) drug efficacy predictors and biomarkers. Other related perturbational databases such as Connectivity Map (CMAP),^[^ ^10^ ^]^ the Library of Integrated Network‐Based Cellular Signatures (LINCS‐L1000),^[^ ^11^ ^]^ and Cancer Therapeutics Response Portal (CTRP)^[^ ^12^ ^]^ also include numerous compound sensitivity characteristics of cell lines for drug perturbation analysis. Those large‐scale pharmacogenomic datasets offer researchers an opportunity to better characterize the heterogeneity of perturbational biological features through machine learning technologies.^[^ ^13^ ^]^ For instance, Peilin et al. developed a deep learning model to impute drug responses in both tumor cell lines and cancer patients, demonstrating the clinical translational potential of the deep learning (DL) method for drug response biomarker discoveries.^[^ ^14^ ^]^ Hostallero et al. built a tumor tissue‐informed normalization deep learning (TINDL) model to impute the compound efficacy on tumor patients, which integrated transcriptome profiles of both the GDSC database and TCGA primary samples.^[^ ^15^ ^]^ To alleviate the discrepancies between cell lines and tumor tissues, TINDL integrates tumor type information of test samples to enhance the performance of drug efficacy prediction on patients.^[^ ^15^ ^]^ Similarly, Hossein et al. proposed an adversarial inductive transfer learning model for the drug efficacy prediction on four anti‐cancer compounds.^[^ ^16^ ^]^ The adversarial learning is an approach to improve data recognition despite the existence of dataset bias^[^ ^17^ ^]^ and was applied to bridge the gap between cell lines and clinical datasets (TCGA patient, clinical trials) to improve model generalization.^[^ ^16^ ^]^

We noticed that the above models were constructed based on bulk RNA‐seq data and many therapeutic strategies of patients are derived from mean recommendations informed by meta‐analysis. The major drawback of bulk RNA‐seq is that it only gives the average and a mixture profile of heterogeneous cells. Its limited capability conceals the uniqueness as well as the actual diversity of individual cells.^[^ ^18^ ^]^ It was suggested that a multitude of cancer cells with distinct molecular characteristics could coexist in the same tissue. In addition, a small proportion of pre‐existing or acquired resistant cells may evolve and gradually weaken the original medical strategy, and ultimately cause treatment failure.^[^ ^19^, ^20^ ^]^ These therapeutic resistances due to tumor heterogeneity suggest that those “one‐size‐fits‐all” evidence‐based medicine approaches, which is informed by bulk RNA‐seq analysis, may provide inappropriate medical solutions for outlier patients.^[^ ^21^ ^]^

It is noteworthy that this “one‐size‐fits‐all” treatment situation is now shifting to an era of precision medicine in many tumors such as breast cancer,^[^ ^22^ ^]^ metastatic colorectal cancer, and non‐small cell lung cancer.^[^ ^23^ ^]^ New diagnostics such as molecular imaging and gene expression quantification,^[^ ^22^ ^]^ as well as novel clinical trial designs, resulting in a reduction of treatment failure and a decreased morbidity rate.^[^ ^23^ ^]^ Scientists are now calling for more precise methodologies for personalized treatments.^[^ ^24^ ^]^ The emergence and development of single‐cell sequencing technology offers us an opportunity to decipher previously undiscovered heterogeneity of cancer,^[^ ^25^ ^]^ facilitating the identification of previously hidden cell subpopulations with distinct characteristics.^[^ ^26^ ^]^ Recently, a variety of machine learning (ML) and statistical methods have been developed for different single‐cell transcriptomics tasks, such as subtype clustering^[^ ^27^ ^]^ and cell type distinction.^[^ ^28^ ^]^ Nevertheless, due to the limited amount of scRNA‐seq data with both sequencing and drug efficacy information (e.g., cell viability assay), there are few ML models focused on single‐cell drug efficacy prediction, especially those cells sequenced prior to drug treatment. The scRNA‐seq is currently more expensive than bulk sequencing, prolonging its application in large‐scale pharmacogenomic research and clinical studies.^[^ ^29^ ^]^ Although the latest released version of Single Cell Portal from Broad Institute includes over four hundred single‐cell studies, few of them contain drug sensitivity data.^[^ ^30^, ^31^ ^]^ Based on the fact that multiple ML models have elevated the drug efficacy prediction performance on tumor patients by integrating transcriptome information from large cell line pharmacogenomic databases,^[^ ^15^, ^16^ ^]^ we hypothesized that the technical shortcoming of scRNA‐seq could be alleviated by integrating the plentiful transcriptome profiles from cell lines of large pharmacogenomic databases while keeping the microscopic resolution features of scRNA‐seq data. In summary, the drug perturbation effect information could be learned and migrated to the scRNA‐seq drug sensitivity prediction task and biomarker discovery.

**Figure 1:**
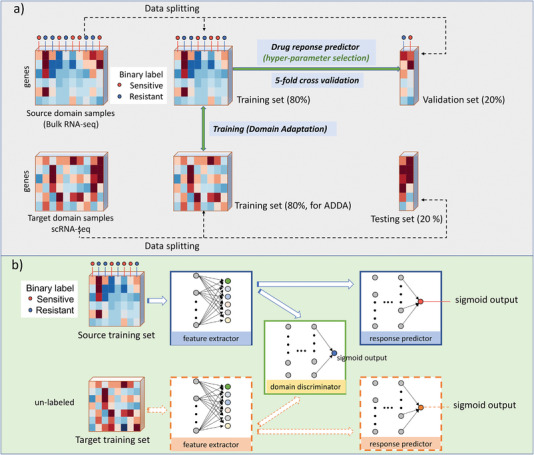
Architecture of SCAD model. a) Data splitting of the source domain and target domain. The SCAD applied a five‐fold cross validation resampling method. For each split, four‐fold of the source domain and target domain are used to train the model, the details of training of process are described in part (b). Another fold of the source domain data is selected as validation set for hyper‐parameter selection. The performance of SCAD is quantified by the average AUC and AUPR scores on the isolated testing set of the target domain. b) Domain adaptation of SCAD, includes a shared feature extractor that extracts the latent feature representations of the source domain and target domain samples; a domain discriminator to help the feature extractor to learn domain invariant features from both the source domain and target domain; a domain communal drug response predictor which was trained by minimizing the loss of the predicted and ground truth drug sensitivities of the source domain data.

In this study, we leveraged transfer learning to learn the pharmacogenomic characteristics of cell lines in the GDSC database to infer the drug sensitivities of samples at the single cell level (**Figure** **1**). The source domain that we extract features is the bulk RNA‐seq from the GDSC database; and the target domain is the scRNA‐seq datasets for the learned model to infer drug sensitivities. Specifically, the drug response predictor was trained based on the expression profiles and binarized response labels (sensitive or resistant) of cell lines from the GDSC dataset. Direct predictions generated by the drug response predictor on scRNA‐seq (as observed in non‐ADDA results of Table 5, Table 6) are tested to exhibit poor performance, due to dataset bias. Such kind of dataset bias is known as domain shift.^[^ ^32^ ^]^ Therefore, we proposed the single cell drug response prediction framework by integrating adversarial domain adaptation (SCAD) to eliminate the domain shift problem. The model adopts the idea of adversarial discriminative domain adaptation (ADDA) composed of both adversarial learning and domain adaptation. The adversarial learning pits two networks against each other.^[^ ^32^ ^]^ In our SCAD framework, the feature extractor and domain (datasets) discriminator are trained in an adversarial manner.^[^ ^32^ ^]^ In the end, the feature extractor can extract domain‐invariant feature representations that the domain discriminator cannot distinguish whether the features come from the source domain or the target domain.^[^ ^32^ ^]^ Moreover, the adversarial learning is an approach to improve data recognition despite the existence of dataset bias.^[^ ^17^ ^]^ Domain adaptation is a method to alleviate the unfavorable effects of dataset bias, reducing the differences between the source and target domain distributions to enhance the model generalization.^[^ ^32^ ^]^ The domain‐invariant feature representations could guarantee the transferability.^[^ ^32^ ^]^ Based on this benefit, we proposed and adopted ADDA to minimize the domain discrepancy between cell lines (source domain) and scRNA‐seq dataset (target domain).^[^ ^32^ ^]^ In addition, we considered transfer learning as two independent tasks for the cells with different tolerant origins based on the mechanism that drug tolerance may occur due to pre‐existing cell populations before treatment or resistance‐acquired cells after drug exposure.^[^ ^20^ ^]^ The details of the source domain and target domain datasets with shared compounds, which are proposed to train a model for drug sensitivity prediction at cells after drug exposure, are provided in **Table** **1**. Similarly, the datasets for the inference of pre‐existing drug‐resistant cells prior to treatment are listed in **Table** **2**.

**Table 1:** scRNA‐seq after drug exposures and corresponding source domain data

Dataset	Drug	Cell line	Type	No. Res	No. Sens	No. Genes
Source domain						
GDSC	Etoposide	Pan‐Can	bulk	811	53	9738
GDSC	PLX4720	Pan‐Can	bulk	746	88	11 937
Target domain						
GSE149215	Etoposide	PC9	10x	764	629	9738
GSE108383	PLX4720	451Lu	SMART‐seq	113	84	11 937
GSE108383	PLX4720	A375	SMART‐seq	46	62	11 937

(Cell line: the cell lines that the bulk RNA‐seq or scRNA‐seq profiled; Type: Sequencing types (bulk: bulk RNA‐seq; 10x: 10x scRNA‐seq; SMART‐seq: SMART‐seq scRNA‐seq); No. Genes: the number of genes that shared by the source domain and target domain. For the source domain dataset, No. Res and No. Sens represent the number of drug‐resistant cell lines and the number of drug‐sensitive cell lines, respectively. For the target domain dataset, No. Res and No. Sens represent the number of drug‐resistant (single) cells and the number of drug‐sensitive (single) cells, respectively.)

**Table 2:** scRNA‐seq prior to drug exposure and corresponding source domain data

Dataset	Drug	Cell line	Type	No. Res	No. Sens	N.A.	No. Genes
Source domain							
GDSC	Gefitinib	Pan‐Can	bulk	714	115	/	10 610
GDSC	Vorinostat	Pan‐Can	bulk	774	60	/	10 610
GDSC	AR‐42	Pan‐Can	bulk	811	80	/	10 610
GDSC	NVP‐TAE684	Pan‐Can	bulk	358	37	/	10 684
GDSC	Afatinib	Pan‐Can	bulk	682	150	/	10 684
GDSC	Sorafenib	Pan‐Can	bulk	362	31	/	10 684
GDSC	Cetuximab	Pan‐Can	bulk	739	122	/	10 684
Target domain							
CCLE	Gefitinib	JUH006	10x	33	33	259	10 610
CCLE	Vorinostat	JUH006	10x	33	33	259	10 610
CCLE	AR‐42	JUH006	10x	33	33	259	10 610
CCLE	NVP‐TAE684	SCC47	10x	60	60	472	10 684
CCLE	Afatinib	SCC47	10x	60	60	472	10 684
CCLE	Sorafenib	SCC47	10x	60	60	472	10 684
CCLE	Cetuximab	SCC47	10x	60	60	472	10 684

(For source domain dataset, No. Res and No. Sens represent the number of drug‐resistant cell lines and the number of drug‐sensitive cell lines, respectively. For target domain dataset, No. Res and No. Sens represent the number of drug‐resistant (single) cells and the number of drug‐sensitive (single) cells, respectively. N.A.: Drug sensitivity label is not available.)

## 2 Results

It was suggested that the resistance to drug treatment can occur due to the growth of the acquired drug‐tolerant cells or the expansion of pre‐existing subclones.^[^ ^19^ ^]^ In this study, we evaluated the prediction performance of our SCAD model in two scenarios independently. Due to the unbalanced ratio of sensitive and resistant cell lines in the source domain, we conducted both weight sampling and smote sampling in model training processes (abbreviated as Weight and Smote, respectively). In addition, we considered three different strategies to explore the impact of performance in the feature selection step before training, namely “all” (which includes all genes shared by source domain and target domain), “tp4k” (the top four thousand highly variable genes in scRNA‐seq by the function of *highly_variable_genes* of Scanpy, which is a simple but popular method for denoising and feature selection strategy prior to downstream analysis on scRNA‐seq data^[^ ^33^ ^]^), and “PPI” (a subset of 2128 protein–protein interaction genes, a prior knowledge that is considered as the most informative gene set for drug sensitivity prediction^[^ ^34^ ^]^).

In this study, we relied on average AUC and average AUPR scores as the performance metrics. We also applied a ranking‐based strategy to sort single cells from the inferred most positive (drug‐sensitive) cells to the most negative (drug‐resistant) one for cell stratification and biomarker exploration. Interestingly, we observed that drug‐resistant cell subpopulation to a specific compound may be sensitive to other drugs. This phenomenon offers us an opportunity to explore and identify promising drug pairs for drug‐combination.

### 2.1 Drug Sensitivity Prediction in Drug‐Tolerant Cells after Treatment

Patients may suffer from drug tolerance within a short term due to the evolution of acquired drug‐resistant cells after treatments.^[^ ^19^ ^]^ Therefore, parental cells (sequenced from drug untreated cell lines) are regarded as sensitive cells, while drug‐tolerant cells that survived after drug exposures are selected and considered as resistant cells.^[^ ^35^ ^]^ We conducted transfer learning on two GSE datasets with both scRNA‐seq profiles and drug response experiments from three different cell lines (see datasets of Experimental Section). The means and standard deviations (SD) of the AUC and AUPR scores for each drug are summarized in Table S1, Supporting Information. We observed that both two metrics under “all” and “tp4k” feature selections were increased after domain adaptation, comparing to two baseline (non‐ADDA) models (**Tables** **3** and **4**). Among all three feature selection strategies, smote sampling which consists of all genes shared by the source domain and target domain obtained the highest prediction results for Etoposide (Smote_all: average AUC = 0.694, average AUPR = 0.736, (Tables 3, and 4). As for the prediction on scRNA‐seq from 451Lu cell line after PLX4720 treatment, we observed that both average AUC and average AUPR scores are close to 0.5 (Weight_tp4k and Smote_tp4k), more like a random prediction. In contrast, better performance values (Smote_all: average AUC = 0.825, average AUPR = 0.875; Smote_tp4k: average AUC = 0.937, average AUPR = 0.948) were achieved in scRNA‐seq from A375 cell line after PLX4720 treatment, comparing to 451Lu cell line (Table 3 and 4; Table S1, Supporting Information). The distinct prediction performance of the same drug PLX4720 in the two cell lines suggests that other confounding factors exist in the drug sensitivity inference. In the study, the researchers identified biomarkers of PLX4720 (BRAFi) resistance in melanoma single cells; 451Lu and A375 cell lines were found to have distinct transcriptome profiles.^[^ ^35^ ^]^ In addition, they clustered the gene expression profiles of 451Lu_BR (BRAFi resistant), 451Lu_Par (parental, drug untreated), A375_BR (BRAFi resistant), and A375_Par (parental, drug untreated) cells to observe the expression patterns among those BRAFi treated and untreated cells. They found that the untreated 451Lu cells are clustered with BRAFi‐resistant 451Lu cells. Similarly, the untreated A375 cells are clustered with BRAFi‐resistant A375 cells.^[^ ^35^ ^]^ The clustering result suggested that the cell type diversity could be more prominent than the drug perturbational effect (The clustering result can be seen in Figure 3c of ref. [35].) Such phenomenon partially explains the distinct performance for drug PLX4720 between A375 cells and 451Lu cells (Tables 3 and 4).

**Table 3:** Average AUC scores of SCAD among three different feature selection strategies (post‐treat)

Drugs	Weight_all (non‐ADDA)	Smote_all (non‐ADDA)	Weight_all	Smote_all	Weight_tp4k	Smote_tp4k	Weight_PPI	Smote_PPI
Etoposide	0.566	0.546	0.669	**0.694**	0.583	0.671	0.500	0.544
PLX4720 (451Lu)	0.450	0.334	0.381	0.381	**0.508**	0.504	0.382	0.354
PLX4720 (A375)	0.727	0.845	0.694	0.825	0.78	**0.937**	0.767	0.798
Average	0.581	0.575	0.581	0.633	0.624	**0.704**	0.550	0.565

(Weight: weight sampling; Smote: smote sampling; all: all genes shared by source domain and target domain, tp4k: the top four thousand highly variable genes in scRNA‐seq, a simple but popular method for denoising and feature selection prior to downstream analysis on scRNAseq datasets,^[^ ^36^ ^]^ and PPI: a subset of 2128 protein‐protein interaction genes described in a recent study.^[^ ^34^ ^]^)

**Table 4:** Average AUPR results of SCAD among three different feature selections (post‐treat)

Drugs	Weight_all (non‐ADDA)	Smote_all (non‐ADDA)	Weight_all	Smote_all	Weight_tp4k	Smote_tp4k	Weight_PPI	Smote_PPI
Etoposide	0.450	0.582	0.694	**0.736**	0.588	0.688	0.55	0.578
PLX4720 (451Lu)	0.511	0.424	0.478	0.461	**0.535**	0.531	0.453	0.429
PLX4720 (A375)	0.793	0.875	0.769	0.875	0.805	**0.948**	0.799	0.861
Average	0.584	0.627	0.647	0.691	0.643	**0.722**	0.601	0.623

### 2.2 Drug Sensitivity Prediction for Pre‐Existing Drug‐Resistant Cells before Treatment

#### 2.2.1 Evaluation of SCAD Based on Source Domain from All Cancer Types

As mentioned in our introduction, several studies have evaluated single‐cell data on selection pressure following drug treatment.^[^ ^5^, ^6^ ^]^ However, there are limited research that evaluate the feasibility of drug response prediction on single cells prior to drug treatment using machine learning. Therefore, we focus more on exploring the feasibility of machine learning in predicting single‐cell drug sensitivities before drug treatment. It was suggested that co‐existing distinct subpopulation prior to drug exposure in cancer may respond differently to drug treatments, facilitating treatment failure, and disease progression.^[^ ^18^ ^]^ Albeit an effective response may occur within a short time after drug exposure, the growth of pre‐existed intratumoral drug‐resistant subclones will eventually cause tumor progression or lethal outcome.^[^ ^2^, ^19^ ^]^ Therefore, successfully deciphering the intratumoral heterogeneity before drug treatment, which is more probable to be achieved by scRNA‐seq than bulk RNA‐seq due to technical advantage, may give informative references for biomarker discovery and treatment decision making. To address this, we conducted several experiments on single cells from SCP542 project. The SCP542 is a single cell portal that contains the sequencing profile of 53 513 cells across 22 cancer types from 198 cell lines, which aims to uncover recurrent cellular heterogeneity on pan‐cancer (22 cancer types) scale.^[^ ^20^ ^]^ Among them, there are around 1000 single cells prior to drug treatment that sequenced from two different cell lines (Table 2) with drug sensitivity information. More specifically, these cells contain the drug sensitivity information for a total of seven anti‐cancer drugs that shared with GDSC database. Therefore, we leveraged transfer learning on single cells that were sequenced from these two cell lines (Table 2). Cell lines from the GDSC dataset with both bulk RNA‐seq profiling and in vitro cell viability assay information were selected as source domain data (Table 2). The detail of data pre‐processing is described in Experimental Section.

In the discovery of above SCP542 study, researchers identified an epithelial senescence‐related (EpiSen) program that can reflect co‐existing distinct subsets of cells observed within cell lines and tumors.^[^ ^20^ ^]^ The EpiSen program is associated with a secretory phenotype and low level of cell proliferation.^[^ ^20^ ^]^ The EpiSen‐high and EpiSen‐low cells were isolated by fluorescence‐activated cell sorting (FACS), based on the expression level of proteins‐coding genes Claudin4 and AXL.^[^ ^20^ ^]^ By performing the in vitro cell viability assay on the FACS sorted cells with different EpiSen program levels in two head and neck squamous cell carcinoma (HNSCC) model cell lines (JHU006 and SCC47), the authors demonstrated that the EpiSen‐high and EpiSen‐low cells derived from same cell lines show different responses to a dozen of drugs.^[^ ^20^ ^]^ The authors generated a method to computationally define the EpiSen program status for every single cell, where cells could be classified into EpiSen‐high (cells with EpiSen score within the upper 10% high among all cells), EpiSen‐low (cells with EpiSen score within the bottom 10% low among all cells), and a third neutral reference population (the detail of calculation could be found in “defining program scores in each cell” method in the previous study^[^ ^20^ ^]^). Inspired by the above work, the scRNA‐seq profiles sequenced from the JHU006 cell line and the SCC47 cell line are treated as target domains in our SCAD transfer learning task. GDSC cell lines with both bulk RNA‐seq profiles and drug sensitivity labels from the in vitro experiments^[^ ^9^ ^]^ were selected to evaluate whether the transcriptome feature and pharmacogenomic knowledge from the source domain could be learned and transferred to help better identify the distinct single cell subpopulations with diverse drug sensitivities prior to treatment.^[^ ^20^ ^]^ The details of the source domain and the target domain are tabulated in Table 2.

As demonstrated in **Tables** **5** and **6**, both metrics under “all” feature selection were increased after domain adaptation, comparing to two baseline (non‐ADDA) models. The means and standard deviations of the AUC and AUPR scores for each drug are summarized in Table S1, Supporting Information. In addition, among all seven compounds, three drugs (Gefitinib, AR‐42, and Cetuximab) got the top mean AUC and AUPR scores under Weight_all sampling method. Four drugs (Vorinostat, NVP‐TAE684, Afatinib, and Sorafenib) obtained the top performance in both metrics under Smote_all sampling method. In addition, among all seven compounds, Weight_all obtained the highest average AUC (0.831) and AUPR (0.850) scores, compared to the rest of the five methods. In contrast, none of the drugs got the highest metrics under PPI or tp4k feature selection, no matter by weight sampling or smote sampling.

**Table 5:** Average AUC results of SCAD among three different feature selections (prior to treat)

Drugs	Weight_all (non‐ADDA)	Smote_all (non‐ADDA)	Weight_all	Smote_all	Weight_tp4k	Smote_tp4k	Weight_PPI	Smote_PPI
Gefitinib	0.888	0.886	**0.967**	0.773	0.862	0.685	0.767	0.487
Vorinostat	0.877	0.685	0.902	**0.942**	0.827	0.826	0.805	0.615
AR‐42	0.568	0.922	**0.968**	0.801	0.784	0.854	0.775	0.737
NVP‐TAE684	**0.744**	0.699	0.598	0.654	0.651	0.564	0.556	0.481
Afatinib	0.684	0.733	0.880	**0.925**	0.746	0.878	0.564	0.547
Sorafenib	0.737	0.696	0.582	**0.771**	0.739	0.666	0.609	0.620
Cetuximab	0.812	0.666	**0.923**	0.798	0.854	0.648	0.638	0.582
Average	0.759	0.755	**0.831**	0.809	0.780	0.732	0.673	0.581

**Table 6:** Average AUPR results of SCAD among three different feature selections (prior to treat)

Drugs	Weight_all (non‐ADDA)	Smote_all (non‐ADDA)	Weight_all	Smote_all	Weight_tp4k	Smote_tp4k	Weight_PPI	Smote_PPI
Gefitinib	0.909	0.915	**0.973**	0.824	0.907	0.755	0.833	0.587
Vorinostat	0.869	0.723	0.904	**0.958**	0.830	0.873	0.828	0.708
AR‐42	0.635	0.928	**0.970**	0.826	0.798	0.894	0.810	0.785
NVP‐TAE684	**0.766**	0.719	0.664	0.695	0.664	0.638	0.624	0.534
Afatinib	0.730	0.758	0.889	**0.938**	0.794	0.912	0.617	0.629
Sorafenib	0.744	0.720	0.627	**0.794**	0.753	0.703	0.656	0.674
Cetuximab	0.838	0.731	**0.921**	0.843	0.883	0.711	0.686	0.649
Average	0.784	0.785	**0.850**	0.840	0.804	0.784	0.722	0.652

As mentioned above, among all seven drugs that were evaluated (sequenced before treatment), considering all genes shared by the source domain and the target domain obtained higher five‐fold average AUC and AUPR scores than merely considering tp4k highly variable genes or PPI genes (Table 5 and 6, Table S1, Supporting Information). One possible reason is that the inclusion of more genes could offer more information for the domain discriminator to force the feature extractor to better learn the domain‐invariant features from both domains, considering the high dropout events in single cell sequencing.^[^ ^36^ ^]^ In this way the drug response predictor trained in the source domain could well predict the drug sensitivities in the target domain. As an example of understanding and visualizing the domain adaption process, we conducted the t‐SNE technique, which is a dimensional reduction method to visualize high dimensional data in 2D or 3D,^[^ ^37^ ^]^ to visualize the representations learned from feature extractor (Weight_all) in drug AR‐42 with the highest average AUC value under Weight_all mode (Table 5). We found that at the very beginning (epoch = 1) of the training process, the extracted features of drug‐sensitive and resistant samples from both domains are mingled (Figure S1, Supporting Information). At the end of training (epoch = 40) after the domain adaptation, the latent features of drug‐sensitive samples of the source domain and the target domain are closer than the one before training. Similarly, the t‐SNE embedding of drug‐resistant samples of the source domain and the target domain is closer than the embedding before training (Figure S1, Supporting Information). Therefore, the feature extractor and domain discriminator together enable the model to learn the invariant features for drug sensitivity classification in the target domain.

#### 2.2.2 Evaluation of SCAD When considering the Cell Line Lineage Origins on the Source Domain

**Figure 2:**
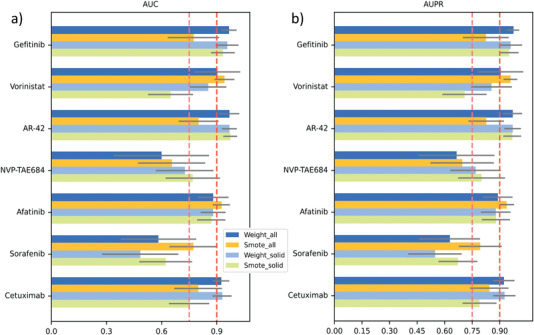
Evaluation of SCAD when the source domain includes all cell lines and when the source domain includes only solid tumor cell lines. a,b) Average AUC and AUPR scores of SCAD in seven drugs, respectively. The scRNA‐seq profiles were sequenced prior to drug treatment.

It was suggested that solid tumor cell lines and hematopoietic cell lines show distinct drug responses.^[^ ^14^ ^]^ However, the impact of source domain cell line origins on the prediction performance of SCAD in single cell data is unknown. To address it, we retained cell lines of solid tumor origin (which is the majority of cancer type in GDSC database) while excluding hematopoietic cell lines (blood cancer) derived from leukemia, lymphoma, and myeloma cancer. The sample sizes of GDSC source domain cell lines for each compound with different tumor type origins are listed in Table S3b, Supporting Information. The t‐SNE plots for the distribution of drug‐sensitive cell lines and drug‐resistant cell lines as well as the corresponding cancer types (solid or blood cancer) can be found in Figure S2a,b, Supporting Information. The drug targets and the mechanism of actions (MoAs) for each drug are summarized in Table S2, Supporting Information. For all seven drugs that we included in our analysis for drug sensitivity prediction on single cells prior to drug treatment, three of them (Gefitinib, Afatinib, and Cetuximab) target EGFR signaling pathway; two drugs (NVP‐TAE684, Sorafenib) target RTK signaling pathway; and two drugs (Vorinostat, AR‐42) are histone deacetylase (HDAC) inhibitors that target chromatin histone acetylation (https://www.cancerrxgene.org/). We conducted a fisher exact test for the frequency distribution of drug‐sensitive cell lines and drug‐resistant cell lines between solid tumor origin and hematopoietic origin in the source domain for each compound. Among all seven drugs, six of them have a significantly different (*p* <  0.01) frequency distribution between solid tumor origin cell lines and hematopoietic ones (Table S3b and Figure S2, Supporting Information), which is identical to the previous study that the cancer type (solid tumor vs blood cancer) will influence drug treatment efficacy.^[^ ^14^ ^]^ Since our previous results showed that when all genes were included, the model performed better than only tp4k high variable genes and PPI genes were considered (Tables 5 and 6). Therefore, we directly compared the prediction performance of SCAD when all cell lines and solid‐tumor‐only cell lines were included under Weight_all and Smote_all sampling modes. We noticed that the performance of our SCAD model was fluctuated under different drugs and sampling modes. However, there is an interesting observation that when blood cancer cell lines were removed, the performance of the drugs Vorinostat and Sorafenib are decreased under both Weight_all and Smote_all sampling modes (Tables 5 and 6); for instance, under Weight_all sampling, the AUC of Vorinostat is decreased from 0.902 to 0.853 and the AUC of Sorafenib is decreased from 0.582 to 0.483. Under Smote_all sampling, the AUC of Vorinostat is decreased from 0.942 to 0.648 and the AUC of drug Sorafenib is decreased from 0.771 to 0.622 (**Figure** **2**; Table S4, Supporting Information). The AUPR and AUPC scores of these two drugs have a similar decreasing effect.

The above results can be attributed to the underlying mechanisms of actions (MoAs) of the drugs (Table S2, Supporting Information), suggesting that drugs may respond differently between solid cancer and blood cancer; for instance, the proportion of Vorinostat‐sensitive cell lines in solid tumor is 2.79% (19/680), while a significant higher proportion (41/154, 26.62%) of sensitive ones was observed in hematopoietic cell lines (Fisher's exact test: *p* < 1 × 10^−5^). It was supported with clinical evidence that blood cancers are especially responsive to HDAC inhibitors, and Vorinostat is one of the promising options for blood cancers with manageable drug‐related adverse events.^[^ ^38^ ^]^ Moreover, Vorinostat was approved by US Food and Drug Administration (FDA) to treat advanced cutaneous T‐cell lymphoma (CTCL).^[^ ^38^ ^]^ Similarly, there is a different proportion of Sorafenib‐sensitive cell lines between solid cancer and blood cancer (10/251, 3.98% vs 21/142, 14.79%; Fisher's exact test: *p* = 0.0003). Removing the blood cancer cell lines of Sorafenib results in decreased prediction results on single cell data. Sorafenib, a first‐generation type‐II multi‐kinase inhibitor, could interact with multiple cell surface kinases (KIT, FLT‐3, Vascular Endothelial Growth Factor‐2 (VEGFR‐2), and VEGFR‐3).^[^ ^39^ ^]^ It was reported in a recent clinical trial (SORAML, a randomized placebo controlled double blind clinical trial that conducted in Germany) that Sorafenib could led to a significant prolongation of event‐free survival (EFS) and relapse‐free survival (RFS) in patients with newly diagnosed acute myeloid leukaemia (AML, a type of cancer that affects the blood and bone marrow).^[^ ^39^ ^]^ Based on the above results, the exclusion of hematopoietic cell lines for drugs Vorinostat and Sorafenib might result in an insufficient number of sensitive cell lines for the model to learn, resulting in reduced prediction performances of SCAD in single cell data. Therefore, the tumor cell line origin information can enhance drug efficacy inference, due to the MoA differences between solid cancer and blood cancer.

### 2.3 Identification of Gene Biomarkers of Drug Sensitivity

Although we have constructed and evaluated the SCAD model for drug efficacy inference, genes that contribute most to the drug sensitivity prediction are still unknown. However, it is vital to find out those biomarkers to help us better understand the biological mechanism of drug response heterogeneity and the potential for clinical application. Fortunately, a recent functional genomics study includes the pre‐treatment gene expression profiles of 40 head and neck squamous cell cancer (HNSCC) patients (GSE65021).^[^ ^20^, ^40^ ^]^ Among them, 14 patients suffered short progression free survival (PFS) less than 5.6 months (short‐PFS group), and 26 patients obtained long PFS exceeding 12 months (long‐PFS group) after following Cetuximab treatment together with a first line platinum‐based chemotherapy.^[^ ^20^, ^40^ ^]^ This GSE65021 dataset can be regarded as an independent clinical cohort to explore the prognostic value of our SCAD model. For a comprehensive comparison with biomarkers identified by bulk RNA‐seq as the evidence‐based support and to evaluate the prognostic prediction value of gene biomarkers that independently identified from scRNA‐seq data, we also selected the drug Cetuximab as our case study.

**Figure 3:**
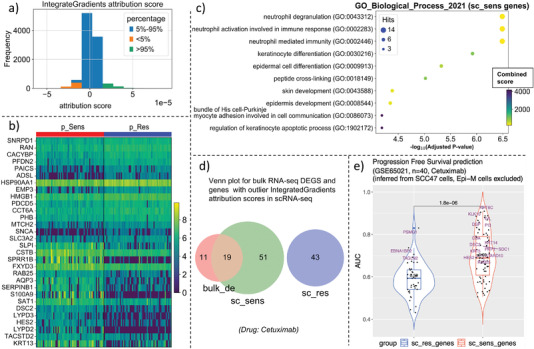
Identification of gene biomarker attributions to drug (Cetuximab) response heterogeneity. a) Density plot for IntegratedGradient attribution score of each gene in the scRNA‐seq expression data of the target domain. b) Heatmap for the top15 up‐regulated genes in p_Sens cells and the top15 up‐regulated genes in p_Res cells. In order to identify high confidence biomarkers, only genes with the largest absolute IntegratedGradient attribution scores (the subset of genes whose contribution values fall in the <5% interval or >95% interval in (a)) are retained for differential expression analysis. c) The gene enrichment analysis (Go Oncology Biological Process v2021) for identified candidate drug sensitive‐related genes for compound Cetuximab. d) Venn plot for the overlap of differentially expressed genes that is reported in a previous study^[^ ^20^ ^]^ and differentially expressed genes (p_Sens vs. p_Res cells) among genes with largest absolute IntegratedGradient attribution scores (those genes whose contribution value falls in the <5% interval or >95% interval in (a)) in scRNA‐seq. The bulk_de is the shorthand for the differentially expressed genes from the bulk RNA‐seq result in the previous study;^[^ ^20^ ^]^ sc_sens refers to the predicted gene set that may make cells susceptible to drugs; sc_res refers to the potential gene biomarkers that contribute to drug resistance. e) Progression free survival (PFS) status prediction on GSE65021 cohort based on the expression level of biomarker genes on GSE65021 dataset; the biomarkers are identified from scRNA‐seq data.

First, we adopted IntegratedGradients^[^ ^41^ ^]^ method to infer the subset of genes that contributes the most to drug sensitivity prediction^[^ ^42^ ^]^ (see Experimental Section). The IntegratedGradients is a method to infer how much an independent variable affects the value of the prediction output from the model, which is one of the most widely used ML model explainer to interpret the association between input data and machine learning model outputs.^[^ ^41^ ^]^ A total of 70 genes that over‐expressed in predicted Cetuximab‐sensitive cells are identified (named sc_sens, **Figure** **3**d). We also identified 43 genes that over‐expressed in predicted Cetuximab‐resistant cells (named sc_res, Figure 3d). For ease of visualization, the top15 overly expressed genes in predicted sensitive cells and the top15 overly expressed genes in predicted resistant cells are presented in Figure 3b, showing distinct gene expression patterns between the two cell populations. We conducted a gene set enrichment analysis and literature search to see if these genes are previously reported with important biological mechanisms. The gene set enrichment analysis (GO_Biological_Process_2021^[^ ^43^ ^]^) results suggested that overexpressed genes in predicted sensitive cells (sc_sens) are enriched in several biological processes (Figure 3c). More specifically, all the top three processes are neutrophil‐related (neutrophil degranulation, neutrophil activation involved in immune response, neutrophil mediated immunity). In contrast, overexpressed genes in predicted resistant cells (sc_res) are enriched in biosynthetic‐related processes (cellular biosynthetic process, monophosphate biosynthetic processes) and regulation of apoptotic process (Figure S3, Supporting Information). It was suggested that the purine biosynthetic pathways are enhanced in cancer cells, which are essential components for the proliferation of cancer.^[^ ^44^ ^]^ The Apoptosis plays an important role in the development of tumor carcinoma, and is one of the key factors that associate with the evasion of apoptosis in cancer.^[^ ^45^ ^]^

Next, we compared our identified promising biomarkers (70 sc_sens genes and 43 sc_res genes, Figure 3b,d) with the reported biomarkers in a previous functional genomics study which compares the differential expression genes between short‐PFS (disease progression free survival) and long‐PFS patients following Cetuximab treatment together with platinum‐based chemotherapy.^[^ ^20^ ^]^ Among the 30 EpiSen‐related genes (Figure S12 in ref. [20]) that differentially expressed between short‐PFS patients and long‐PFS patients, 19 of them are shared with our inferred Cetuximab‐sensitive (sc_sens) genes. However, as for our identified sc_res genes (*n* = 43), we could not directly compared to bulk RNA‐seq data since there are no EpiSen‐related over‐expression genes that were reported in short‐PFS patients of Cetuximab (the detail was described in Figure S12 in ref. [20]). To compare the PFS prognostication ability between sc_sens genes and sc_res genes, we conducted the PFS status prediction on the GSE65021 clinical cohort based on the expression level of our identified cellular biomarker genes. Our results demonstrated that the sc_sens genes have higher prognostication capability (a kind of clinical application value) for PFS outcome prediction than the sc_res genes, where the AUC scores for PFS status (short‐PFS or long‐PFS) prediction based the expression level of the sc_sens gene set are higher than the prediction results based on the expression values of the sc_res genes (median AUC: 0.69 vs 0.59, *p* = 1.8 × 10^−6^, Figure 3e). In addition, nine genes (SERPINB1, KRT6C, SPRR1B, KLK10, IVL, SLPI, DSP, JUP, and PI3) obtained AUC scores higher than 0.85 on GSE65021 cohort (Figure 3e), suggesting that these biomarkers have promising clinical potential. Three newly identified sc_res genes (PSMG1, EBNA1BP2, and TAGLN2, comparing to Figure 3e) which are not reported in ref. [20] were also found with PFS (with Cetuximab treatment) status prediction AUC scores higher than 0.7. With much evidence supported, our discovered biomarkers could uncover some drug heterogeneity mechanisms with prognostication potential, offering a reference for future analysis.

### 2.4 Drug Resistance Ranking of Cells

There are two other questions that need to be further explored. First, researchers focused on distinguishing whether a cell line is sensitive to a compound^[^ ^34^ ^]^ or a patient subset could respond to a candidate drug^[^ ^15^ ^]^ in many previous drug response prediction tasks. Treatment recommendations could then be made based on the prediction results. However, one of the main limitation is that these studies were conducted solely based on bulk RNA‐seq data, masking intratumoral heterogeneity. This leads to a result that, albeit a dramatic response may occur within a short time after the exposure to the selected drugs, patients that received recommended therapies may still suffer from drug‐tolerant and disease progression before long due to the existence of drug‐resistant subclones.^[^ ^19^ ^]^ Therefore, identifying drug‐tolerant subclones within a cell line or one tumor sample is also critical for studying the biological mechanism of tumor resistance, which is difficult to be evaluated by bulk RNA‐seq due to technical limitations. Fortunately, scRNA‐seq allows us to capture the transcriptome profile for each cell, offering us a chance to investigate both drug‐resistant and drug‐sensitive cells simultaneously,^[^ ^18^ ^]^ which is important for the analysis of tumor heterogeneity and decision‐making of precision therapy.

**Figure 4:**
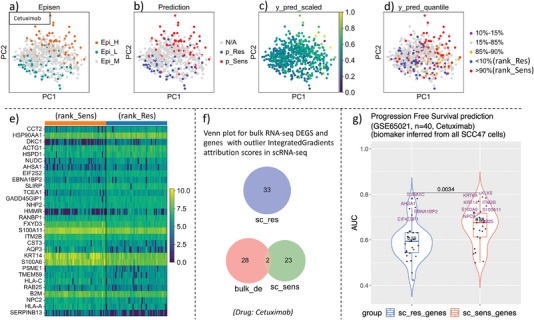
Drug resistance ranking of cells identifies potential prognosis biomarkers. a) The PCA dimensional reduction plot of single cells that is colored by the EpiSen status after the binarization of the EpiSen score, which was regarded as the ground truth drug sensitivity statues of cells to generate the labels of our target domain datasets. b) The PCA plot of single cells that is colored by the binarized (cutoff = median) SCAD prediction values. c) Visualization of single cells by PCA which is colored by the scaled SCAD prediction value after MinMaxScaler. d) Visualization of single cells by PCA plot, in which the SCAD prediction values of cells are stratified and colored by percentiles. e) Heatmap for the top15 up‐regulated genes in rank_Sens cells and the top15 up‐regulated genes in rank_Res cells. Only genes with the largest absolute IntegratedGradient attribution scores (the subset of genes whose contribution values fall in the <5% interval or >95% interval among all genes, abbreviated as outlier genes) are retained for differential expression analysis. f) Venn plot for the overlap of differentially expressed genes that is reported in a previous study (ref. [20]) and differential expression genes between rank_Sens and rank_Res cells in scRNA‐seq. The bulk_de is the shorthand for differentially expressed genes from the bulk RNA‐seq result in the previous study (ref. [20]); sc_sens refers to the predicted gene set that may make cells susceptible to drugs; sc_res refers to the potential gene biomarkers that contribute to drug resistance. g) Progression free survival (PFS) status prediction on GSE65021 cohort based on the expression level of biomarker genes from scRNA‐seq. Biomarkers are inferred from all SCC47 cells. The top 10% most Cetuximab‐sensitive cells and the top 10% most Cetuximab‐resistant cells are selected for differential expression analysis and biomarker inference.

Second, the EpiSen program scoring may be insufficient to capture the whole drug sensitivity profile within the tumor. For instance, the EpiSen program is varied among different tumors since the variance of the EpiSen score is small in many cancer types, such as bone cancer, neuroblastoma, and sarcoma^[^ ^20^ ^]^ (Figure S4, Supporting Information). In addition, the drug sensitivity information is only accessible in those EpiSen‐high and EpiSen‐low cells that have been tested in cell viability assay, and only several drugs have been tested.^[^ ^20^ ^]^ Therefore, the drug perturbation information for cells with moderate EpiSen score (10–90% quantile of all cells) or drugs that have not been evaluated in cell viability assay is not available (Table 2 and **Figure** **4**a). Moreover, the EpiSen program score is an indicator based on the expression level of proteins Claudin4 and AXL. However, some drugs are sensitive in EpiSen‐high cells, while other drugs may be sensitive in EpiSen‐low cells and resistant in EpiSen‐high cells (Table S3, Supporting Information, **Figure** **5**a,b,f,j; Figure S5, Supporting Information). Therefore, we cannot directly infer the drug sensitivity of a given drug based on the binary EpiSen status before conducting in vitro drug experiments. These limitations suggest that the EpiSen program may not be a pan‐cancer drug sensitivity indicator. In contrast, in our source domain, the GDSC database contains transcriptome profiles and drug perturbation information in around 1000 cell lines with over 250 compounds, giving us the opportunity to directly infer drug efficacy on unseen tumor cells.^[^ ^34^ ^]^

**Figure 5:**
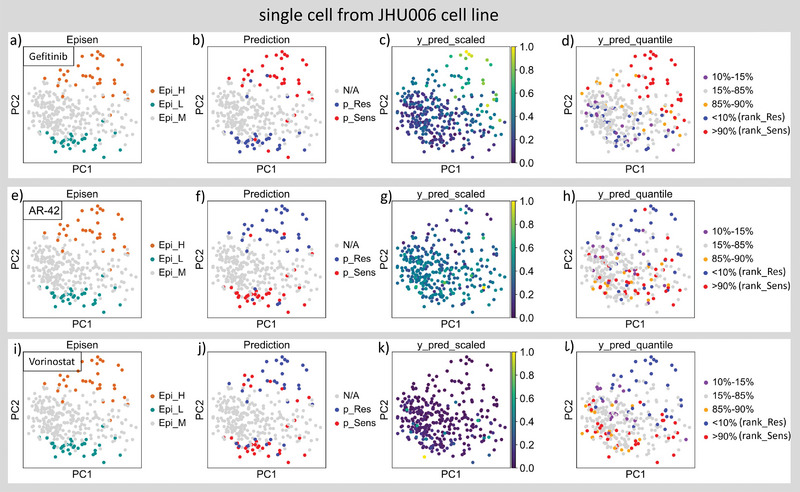
Drug resistance ranking reveal potential drug combinations. a,e,i) The PCA plots of single cells that is colored by the ground truth label after the binarization of the EpiSen score. b,f,j) The PCA plots of single cells that is colored by the binarized (cutoff = median) SCAD prediction values. c,g,k) Visualization of single cells by PCA dimensional reduction which colored by the scaled SCAD prediction value after MinMaxScaler. d,h,l) Visualization of single cells by PCA dimensional reduction, in which the SCAD prediction values of cells are stratified and colored by percentiles.

To evaluate whether our SCAD model could identify drug‐sensitive cells and drug‐resistant ones within tumors, we selected drug Cetuximab, which obtained the highest AUC and AUPR scores under Weight_all strategy among drugs in the SCC47 cell line (Tables 5 and 6). The EpiSen‐high (Epi_H), EpiSen‐moderate (Epi_M), and Episen‐low (Epi_L) cells are colored in yellow, gray, and blue, respectively (Figure 4a). Different EpiSen program states correspond to different drug sensitivities, depending on the in vitro drug response experiments that conducted in previous research (only Epi_H and Epi_L cells are tested in vitro).^[^ ^20^ ^]^ The EpiSen statuses are regarded as drug sensitivity ground truth labels. As a comparison, the corresponding prediction results of known label cells (either Epi_H or Epi_L) are presented in Figure 4b, which are grouped into two cell populations (classified by the cutoff of the median of the output value of drug predictor) and colored in red and blue, respectively. As we can see, the relative drug sensitivity of the majority of cells of known sensitivity in both cell lines was accurately predicted.

Next, to evaluate the prediction results of the SCAD model in all SCC47 cells (Epi_H, Epi_M, Epi_L, Figure 4c,d), we retrained the SCAD for drug Cetuximab. Similar to EpiSen program scoring, the predicted output values of SCAD were then extracted and sorted. We highlighted cells with the top ten percent (>90%, abbreviated as rank_Sens) quantile high and the ten percent bottom low (<10%, abbreviated as rank_Res) of SCAD predicted values (Figure 4d). In addition, to better visualize the distribution of the drug sensitivity profiles determined by the EpiSen program and the drug sensitivity profiles inferred by our SCAD transfer learning model, we slightly relaxed the threshold into the first and last fifteen percent of SCAD predicted values.

The predicted Cetuximab sensitive cells (rank_Sens) and predicted high resistant (rank_Res) cells are relatively distributed at distinct locations in the PCA plots for most cells (Figure 4d), suggesting that there may exists gene expression heterogeneity between Cetuximab‐sensitive cells and Cetuximab‐resistant ones. This phenomenon offers us an opportunity to identify the subset of cells that may cause treatment failure and the subset of cells that are drug sensitive, which is vital for biomarker discovery and treatment decision‐making.

To explore the possible gene expression heterogeneity between predicted Cetuximab‐sensitive cells and Cetuximab‐resistant cells (based on our ranking result), we conducted a similar biomarker exploration process that we introduced in Figure 3. We observed that there still exists a gene expression heterogeneity among predicted Cetuximab‐sensitive (rank_Sens) cells and predicted Cetuximab‐resistant (rank_Res) cells (Figure 4e). This time, we identify 33 genes that over‐expressed in predicted rank_Res cells and 30 genes that over‐expressed in predicted rank_Sens cells (Figure 4e). Although the overlap with previously reported 30 drug sensitive genes was low (two genes, Figure 4f), we observed that our inferred drug sensitivity biomarkers still contain significant different tumor progression free survival (PFS) prognostication ability on GSE65021 dataset (median AUC: 0.68 vs 0.59, *p* = 0.0034, Figure 4g). In addition, three novel drug‐resistance biomarkers (TUBA1C, AHSA1, and EIF4EBP1, comparing to Figure 3e) with PFS status prognostification AUC higher than 0.7 were identified (Figure 4g). This result suggests that our SCAD model could independently capture the drug response heterogeneity and identify promising gene biomarkers, without knowing the EpiSen program status.

### 2.5 Drug Resistance Ranking and Drug Combination

As mentioned above, the EpiSen status is an indicator based on the expression levels of protein‐coding genes Claudin4 and AXL. However, the drug sensitivities of different cell subpopulations cannot be judged from the EpiSen status along unless validated in vitro. As we can see in Figure 5a,e, the Epi_H JHU006 cells are Gefitinib‐sensitive while Vorinostat‐resistant (see also Table S3a, Supporting Information). Similarly, the Epi_H SCC47 cells are Cetuximab‐sensitive while Sorafenib‐resistant (Figure S5c,d and Table S3a, Supporting Information). In our drug resistance ranking result, we found that most of predicted Vorinostat‐resistant (rank_Res) JHU006 cells are predicted as Gefitinib‐sensitive (rank_Sens) cells. In addition, the PCA projection result suggests that the position of Gefitinib rank_Res cells are close to the position of Vorinostat rank_Sens cells. This result suggests that the combination of these two drugs may able to counteract or mitigate drug resistance in different cell subsets. The treatment efficacy of the combination of Gefitinib and Vorinostat has been reported in many studies in clinical and in vitro. For instance, researchers found that the Gefitinib plus Vorinostat has a synergistic effect in inhibiting the growth of head and neck cancer (HNC) cells.^[^ ^46^ ^]^ In addition, the efficacy of this combination is independent on the HPV status, a strong prognosis biomarker in HNC cancer.^[^ ^46^ ^]^ Gefitinib is an epidermal growth factor receptor (EGFR) tyrosine kinase inhibitor (TKI) while Vorinostat is a Histone deacetylase inhibitor (HDACi). The synergistic efficacy of dual EGFRi and HDACi in overcoming resistance to monotherapy was also found in many other cancer types, such as non‐small‐cell lung cancer (NSCLC)^[^ ^47^ ^]^ and mucoepidermoid carcinoma.^[^ ^48^ ^]^ Interestingly, a similar opposite drug response status was found between drug Gefitinib and drug AR‐42 (Figure 5d,e). In addition, AR‐42 is also a HDAC inhibitor, which was reported with significant potency to inhibit the growth of esophageal squamous cell carcinoma (ESCC) cells. Although has not been explored, the synergistic efficacy of dual EGFRi and HDACi suggests that Gefitinib and AR‐42 may be able to reduce the drug resistance of drug Gefitinib. This result should be explored further in future study.

## 3 Conclusion

It is necessary to infer the drug sensitivities of cells and explore the tumor heterogeneity at single‐cell resolution. Previous studies suggested that the anti‐cancer treatment may fail due to the pre‐existing resistant cells prior to treatment or the acquired tolerant ones after drug exposure.^[^ ^49^ ^]^ To address it, many studies have been conducted to collect the tremendous transcriptome sequencing data and drug efficacy information (cell viability assay) of cancer cell lines from large pharmacogenomic databases to infer the drug sensitivities of other cell lines or patient samples.^[^ ^14^, ^15^ ^]^ However, most of them are based on bulk RNA‐seq data, which only give an average profile of cell mixtures, masking cellular heterogeneity.^[^ ^18^ ^]^ Fortunately, the growth of scRNA‐seq data offers an opportunity to elucidate the intratumoral heterogeneity at cellular resolution. There are currently some studies that evaluate the selective pressure after drug treatment using single‐cell proteomics/phosphoprotomics data to study the drug‐induced acquired drug tolerance mechanism and identify possible therapeutic target to address post‐treatment resistance.^[^ ^6^ ^]^ These results suggest the possibility and necessity of conducting additional explorations of drug response heterogeneity in single‐cell data.

The relatively scarce drug sensitivity information at single cells can be alleviated by transfer learning. Currently, high‐throughput pharmacogenomic databases such as GDSC and CCLE harbor numerous transcriptome profiles and drug response sensitivity data,^[^ ^8^, ^9^ ^]^ while the disadvantage is that the resolution is not comparable to single‐cell sequencing. On the contrary, scRNA‐seq drug perturbation data were currently limited, hindering the study of machine learning in single‐cell drug sensitivity prediction. Based on the successful application of transfer learning in many real‐world problems,^[^ ^50^ ^]^ we proposed a transfer learning framework to learn pharmacogenomics information from cell lines in large public dataset GDSC and transfers the learned model to infer drug efficacy at the single cell resolution.

In this analysis, we explored and demonstrated the feasibility of predicting drug sensitivities of single cells by transfer learning. In the SCP542 project, researchers found that the EpiSen‐high and EpiSen‐low cells sequenced from two independent cell lines are featured with distinct drug response sensitivities to several compounds, this phenomenon was validated by the in vitro cell viability assay experiments. We integrated this information and the corresponding single‐cell sequencing profiles into a dataset to investigate the feasibility of transfer learning on single‐cell expression profiles. We conducted several experiments to infer the drug sensitivities of cells with distinct EpiSen program status by transfer learning. Our SCAD model attempts to integrate and transfer the pharmacogenomic knowledge from cell lines to infer the drug efficacy at scRNA‐seq data. In addition, we formulated our transfer learning work as two independent tasks. Our results showed that the model's drug sensitivity prediction performance was improved with the help of domain adaptation (ADDA) compared to two non‐ADDA baselines. Surprisingly, we also found that the prediction performance can be fluctuating when predicting the drug sensitivities between parental cells and resistant cells after drug exposures. One possible reason is that the source domain GDSC cell lines are sequenced at the pre‐clinical stage (prior to the exposure to different drugs)^[^ ^9^ ^]^ which may not be suitable to infer the drug sensitivities of single cells after the treatment of anti‐cancer agents ideally, since the expression of genes may change a lot before and after drug exposure. As a comparison, we observed stable prediction performance (see also tables in Supporting Information) among a total of seven anti‐cancer drugs when evaluating the drug sensitivities in single cells that were sequenced from two cell lines prior to anti‐cancer therapy. These results suggest that pre‐clinical cell lines may be more suitable for transfer learning analysis in pre‐clinical single cells, where both the source domain and the target domain were sequenced before drug exposures.

Drug resistance ranking identify promising prognostication biomarkers and drug combination. It is vital to uncover the black‐box mechanism of machine learning model for model interpretation. We applied IntegratedGradients to explore the relationships between input and output of SCAD model. For Cetuximab, we observed a high overlap of genes between the overexpressed genes in long PFS patients and the ones with high IntegratedGradient attribution scores in predicted sensitive cells. For the genes identified that may contribute to drug resistance, we also found evidence from the published literature that supported our gene oncology (GO) pathway enrichment analysis results (Figure S3, Supporting Information). In addition, we observed that different cell subpopulations may be resistant to one drug and sensitive to another, suggesting the possibility and necessity of drug combination therapy. We found that a part of JHU006 cells were inferred to be resistant to HDACi Vorinostat but sensitive to EGFRi Gefitinib. The positive treatment effect of the combination of Gefitinib and Vorinostat has been reported in many in vitro experiment and researchers across several cancer types, supporting our result that ranking‐based cell stratification strategy that uncover possible drug combinations (e.g., Gefitinib plus AR‐42).

Uncovering the varied drug response sensitivities of different cell subpopulations within tumors is important for precision medicine. Based on the wealth of tissue‐sequencing‐based pharmacogenomics information, we explored and interpreted the feasibility of predicting drug response at the single‐cell level prior to drug treatment. Predicting drug sensitivity at the single‐cell level can be promising in uncovering new prognostic biomarkers with higher resolution and possible combination therapies. There are currently a myriad of bulk‐RNA‐seq‐based pharmacogenomics data and unlabeled single‐cell sequencing data. The integration of those valuable un‐labeled data for pharmacogenomic research and application is a promising direction for personalized medicine. Inspired the study that normalizing source domain data with unlabelled target domain data can enhance the transfer learning prediction performance;^[^ ^15^ ^]^ and as an attempt at data analysis, we normalized the source domain cell lines with a dataset (named as SC‐5K dataset) that contain five‐thousand (5K) single cells (SC). We found that our model obtained slightly increased magnitudes in both AUC and AUPR. Moreover, we also found that the variances of AUC and AUPR in ensemble mode (SCAD (SC‐5K non‐adjusted model plus SCAD SC‐5K adjusted model) are smaller than SCAD (SC‐5K non‐adjusted model), suggesting that the performance of ensemble mode will be more stable than the counterparts (Figure S6, Tables S6 and S7, Supporting Information). We believe that this single cell drug inference transfer learning strategy could be easily extended to other pharmacogenomic tasks and biomarker identification. In addition, those promising drug response heterogeneity‐related genes are selected from around 10 000 genes, narrowing the range of candidate gene selection for downstream studies. In summary, our study provides new insights into the diversity of sensitivity and resistance of different cell subpopulations to anticancer drugs.

## 4 Experimental Section

### Datasets

As previously described, the prediction for pre‐existing drug resistant cells prior to treatment and acquired resistant cells after compound exposure was considered as two independent tasks, due to different drug tolerant mechanisms of tumor heterogeneity were treated.^[^ ^19^ ^]^ As listed in Table 2, the source domain datasets were obtained from the GDSC database. The LOBICO (logic optimization for binary input to continuous out) binarized drug response IC50 values, which label cell lines as drug‐sensitive or drug‐resistant to a specific drug in GDSC database, were obtained from Table S5C of a previous study.^[^ ^51^ ^]^ The scRNA‐seq from JHU006 and SCC47 cell line of Broad Institute's single‐cell portal (https://singlecell.broadinstitute.org/single_cell/study/SCP542/) were selected as the target domain for the evaluation of pre‐existing drug‐sensitive and drug‐resistant cells. Drug response sensitivities for each compound in the corresponding cell lines are listed in Table S3a, Supporting Information. The scRNA‐seq data from GSE149215 (GSM4494347, PC9 untreated; GSM4494354, PC9 cell treated with 25 µm Etoposide for 3 days) and GSE108383 (scRNA‐seq for A375 cell line and 451Lu cell line) datasets were selected as target domain for the evaluation of acquired resistant after the exposure of drug Etoposide and PLX4720, respectively. The details of drug names, cell lines, and sample size of drug resistant and sensitive cells for source and target domain are shown in Table 1 and Table 2.

### Data Preprocessing

The GDSC RMA normalized bulk RNA‐seq data was downloaded as the source domain data, which was processed and described in https://ibm.ent.box.com/v/paccmann‐pytoda‐data/folder/91702227426.^[^ ^34^ ^]^ Genes were standardized into *z*‐score by scikit‐learn StandardScaler function. Next, cell lines with both RMA normalized bulk RNA‐seq expression profiles and LOBICO binarized drug response IC50 values^[^ ^51^ ^]^ were kept for further analysis.

In the SCAD model, scRNA‐seq datasets were regarded as the target domain. Several data quality control (QC) steps described in ref. [36] were followed. As suggested, both cell QC and gene QC are two factors that should be considered.^[^ ^36^ ^]^ More specifically, the quality control pre‐processing steps were conducted using the Scanpy package^[^ ^33^ ^]^ based on the profiling feature of each dataset (includes min_genes, min_counts, min_cells, and max_counts), considering the diversity of technical and sequencing depth among different target datasets (Table 1 and Table 2) 2. In addition, cells with a fraction of mitochondrial counts higher than 20% of molecular counts were also removed.^[^ ^36^ ^]^ The total molecular counts over all genes for each cell were then normalized (e.g., target_sum=1 × ~10^4^ for GSE149215 and GSE108383; and counts per million for SCP542 dataset, which has been preprocessed by data contributor^[^ ^20^ ^]^), log‐transformed and standardized as *z*‐score. In addition, only genes in both the source domain and the target domain were retained for model training and testing.

### Workflow Overview of SCAD

Inspired by the framework from a previous study that used transfer learning to learn pharmacogenomics knowledge from cell lines to predict the drug responses on patients,^[^ ^16^ ^]^ the SCAD model consisted of a feature extractor to learn the representations of the source domain and target domain, a drug response predictor to learn the informative features for drug sensitivity inference, and a domain discriminator to make the feature extractor to learn domain invariant features from both domains (Figure 1).

The source domain data were bulk RNA‐seq of cell lines from the GDSC database with binarized IC50 labels.^[^ ^51^ ^]^ The processed gene expression data was downloaded from a previous cell line drug response prediction research.^[^ ^34^ ^]^ The scRNA‐seq profiles were treated as unlabeled target domain datasets during model training and validation. The testing set of the scRNA‐seq were tested to evaluate the transfer learning performance. A shared feature extractor was constructed and devised to capture the mutual latent information of source domain and target domain. To reduce performance bias due to single data splitting, a five‐fold cross validation resampling procedure was applied in both domains. The drug response predictor implicitly learned the data distribution by relying on the training set of the source domain. The hyperparameters were selected based on the prediction performance on the validation set of the source domain (under five‐fold cross validation strategy). The details of each component of SCAD framework are described as follows and in Figure 1.

### Feature Extractor

The source domain and target domain shared the same feature extractor, enabling weight sharing and symmetric mapping of data from both domains. Similar to ref. [16], the feature extractor was a multi‐layer perceptron (MLP) with dropout rate of 0.5. A hyperparameter h_dim was set to adjust the number of nodes of the feature extractor. The source and target data was denoted as *X*_S_, *X*_T_, and the source and target representation mapping as

(1) $$Z_{s}=M_{s}\left(x_{s}\right),Z_{t}=M_{t}\left(x_{t}\right)$$

### Drug Response Predictor

The drug response predictor was trained solely based on source domain data, and target domain drug sensitivity labels were unseen until final testing. The drug response predictor was a five‐layer MLP with dropout rate of 0.5. The number of nodes in each layer remained the same. A hyperparameter z_dim was set to customize the number of nodes of the hidden layer. A sigmoid function was appended after the five hidden layers of predictor to obtain the drug sensitivity probability. The sigmoid output was denoted as

(2) $${\bar{y}}_{s}=P_{s}\left(Z_{s})\right)=P_{s}\left(M_{s}\left(x_{s}\right)\right)$$

### Domain Discriminator

A domain discriminator (denoted as *D*) was trained to force the feature extractor to learn domain invariant features from both the source domain and target domain. The domain adaptation could help to reduce the difference between the source domain (GDSC bulk RNA‐seq datasets) and the target domain (scRNA‐seq dataset) distributions to enhance the performance of drug response predictor. In this end of training, the feature extractor might be able to extract feature representations (domain‐invariant features) that the domain discriminator was unable to distinguish whether the coming features were extracted from the source domain or the target domain.^[^ ^32^ ^]^ The domain discriminator is a five layer MLP with dropout rate of 0.5. The number of nodes in each layer was the same. A hyperparameter h_dim (hyperparameter that is shared with the feature extractor) was set to customize the number of nodes of the hidden layer. A sigmoid function was appended after the five hidden layers. The domain discriminator outputs for source data and target data were denoted as

(3) $${\bar{d}}_{s}=D\left(M_{s}\left(x_{s}\right)\right),{\bar{d}}_{t}=D\left(M_{t}\left(x_{t}\right)\right)$$

By confusing the discriminator to distinguish whether features from the source domain or target domain and features from the target domain scRNA‐seq data could be mapped by a communal feature extractor (trained with source domain data) to the shared feature space and directly classified by the communal drug response predictor that was learned from the source domain. Solid lines in Figure 1 indicate the source domain modules (source feature extractor, domain discriminator, and drug response predictor). The dashed lines indicate the communal source modules applied to the target domain.

### Loss Function

The performance of the feature extractor and drug predictor were evaluated based on a binary‐cross entropy (BCE) loss

(4) $$\begin{array}{cc} & L_{\text{BCE}}\left(X_{S},Y_{S}\right)=\\ & -\sum_{\left(x_{s},y_{s}\right)\in \left(X_{S},Y_{S}\right)}\left[y_{s}\log{\bar{y}}_{s}+\left(1-y_{s}\right)\log\left(1-{\bar{y}}_{s}\right)\right] \end{array}$$

The adversarial loss for discriminator is

(5) $$\begin{array}{cc} & L_{\text{adv}\;D}\left(X_{S},X_{T},M_{s},M_{t}\right)=\\ & \;-\sum_{x_{s}\in X_{S}}\left[\log D\left(M_{s}\left(x_{s}\right)\right)\right]-\sum_{x_{t}\in X_{T}}\left[\log\left(1-D\left(M_{t}\left(x_{t}\right)\right)\right)\right] \end{array}$$

Referred to ref. [16], a weight λ was introduced to balance the influence of the adversarial loss. Therefore, the total loss of SCAD is

(6) $$L_{\text{scad}}=L_{\text{BCE}}+\lambda L_{\text{adv}D_{G}}$$

### Evaluation Metrics

A five‐fold cross‐validation (CV) strategy was conducted to reduce data splitting bias and evaluate the performance of the SCAD model. In each splitting, four‐fold of the source domain sample were selected as the training set to train the source feature extractor, domain discriminator, and drug response predictor, while another fold of the source domain data was treated as the validation set for hyper‐parameter selection (The final selected hyper‐parameters were tabulated in Table S5, Supporting Information). Similarly, in each splitting, four‐fold of the source domain samples and target domain samples were fed to the shared feature extractor to generate the latent vectors that aim to make the domain discriminator unable to identify whether features were extracted from the source domain or target domain, and one‐fold of target domain sample was tested to evaluate the performance of SCAD model. After hyper‐parameter selection, the model was retrained and evaluated with another four different random seeds to evaluate the comprehensive performance of small sample models with different random seeds. The reported results referred to the average AUC and AUPR over the 25 test folds (five‐folds CV and five random seeds, the testing results are summarized in Tables S1 and S4, Supporting Information). The AUC is a metric to quantify the quality of the model's predictions, measuring how well predictions were ranked. More specifically, it is equivalent to the probability that the classifier will rank a randomly chosen positive example higher than a randomly chosen negative one. Normally, a classifier with a higher AUC suggests a better classification performance than another classifier with a lower AUC. The AUPR calculates the area under the precison‐recall (PR) curve, and the baseline of the PR curve varied in different tasks based on the ratio of positive class rate in whole sample size.

### Identification of Biomarker Genes of Drug Sensitivity with IntegratedGradients

For each fold of the five‐fold cross‐validation, the attribution score for each gene in the testing set of the target domain was calculated and recorded. The attribution scores of each gene were then averaged to get the final mean attribution score, which helped to reduce the bias of single‐fold splitting. Next, genes with the top 5% high or bottom 5% low contribution scores were selected as candidate genes that are related to drug response heterogeneity (Figure 3a). Differential expression analysis by *rank_genes_groups_df()* function was then conducted in Scanpy^[^ ^33^ ^]^ to filter out the inferred statistical significance drug‐resistance and drug‐sensitive related gene.

## Conflict of Interest

The authors declare no conflict of interest.

## Supporting information

Supporting Information

Supplemental Tables S1‐S7

## Data Availability

Datasets and source code are publicly available at https://github.com/CompBioT/SCAD.
