# Supplementary material for: Enabling Single‐Cell Drug Response Annotations from Bulk RNA‐Seq Using SCAD
Source: Adv Sci (Weinh). 2023 Feb 10;10(11):2204113. doi: 10.1002/advs.202204113 (PMC10104628; doi:10.1002/advs.202204113)
Supplement: Supplementary file 1 — Supporting Information [file ADVS-10-2204113-s001.pdf]

ADVANCED SCIENCE
Open Access

Supporting Information

for *Adv. Sci.*, DOI 10.1002/advs.202204113

Enabling Single-Cell Drug Response Annotations from Bulk RNA-Seq Using SCAD

Zetian Zheng, Junyi Chen, Xingjian Chen, Lei Huang, Weidun Xie, Qiuzhen Lin, Xiangtao Li and Ka-Chun Wong**

Supplementary Figures

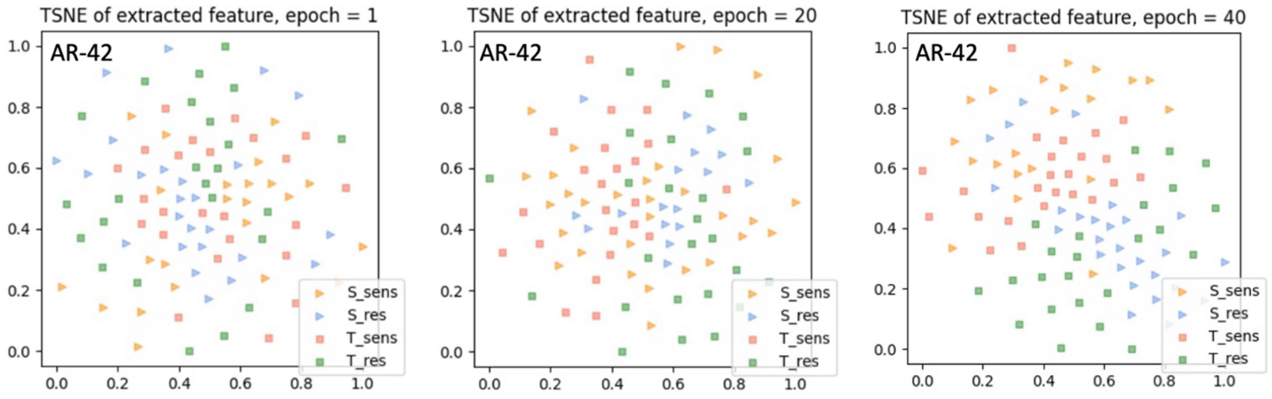

Fig. S1.

The t-SNE plot of extracted latent feature by feature extractor for drug AR-42 at epoch = 1, epoch = 20, and epoch = 40. S_sens, source domain drug sensitive cell lines; S_res, source domain drug resistant cell lines; T_sens, target domain drug sensitive cells; T_res, target domain drug resistant cell.

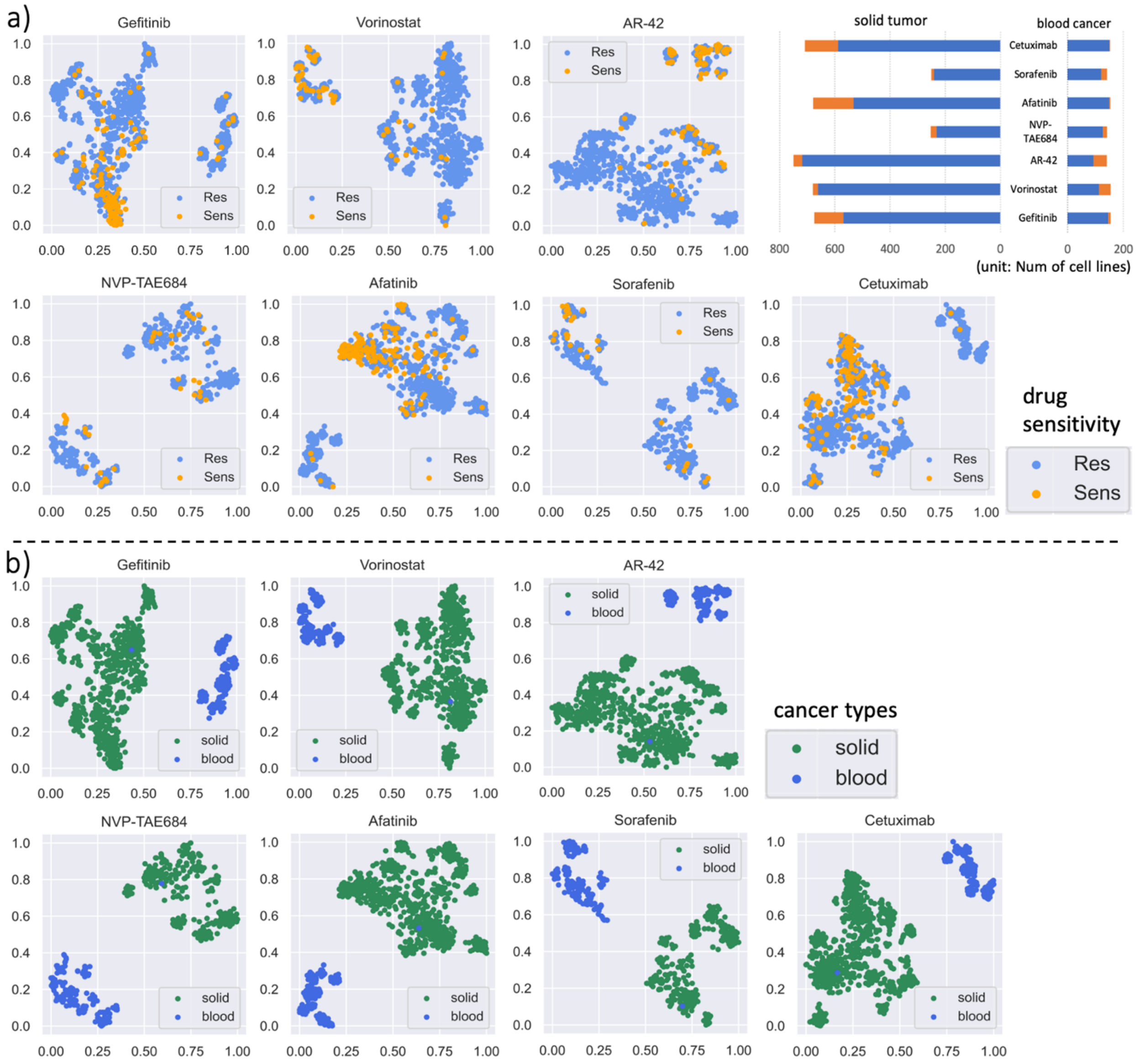

Fig. S2.

The t-SNE plot for the transcriptome profiles of seven drugs that used as source domain to predict the drug sensitivity of single cells before treatment. (a) Cell lines are colored by drug sensitivity (Resistant or Sensitive); (b) Cell lines are colored by cell line lineage origins (solid cancer or blood cancer).

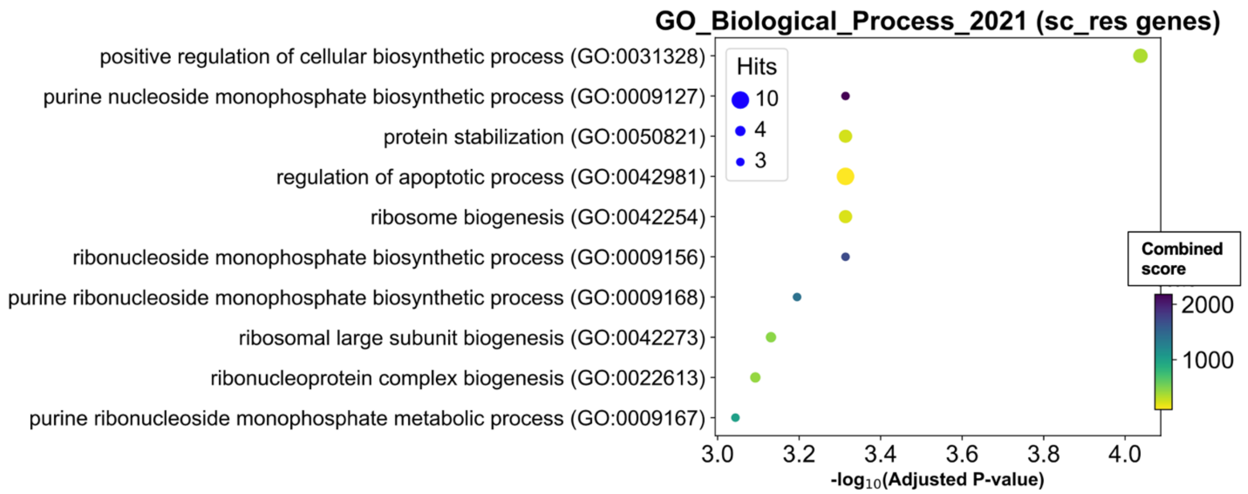

Fig. S3.
The gene enrichment analysis (Go Oncology Biological Progress v2021) for identified candidate drug resistant-related genes for Cetuximab.

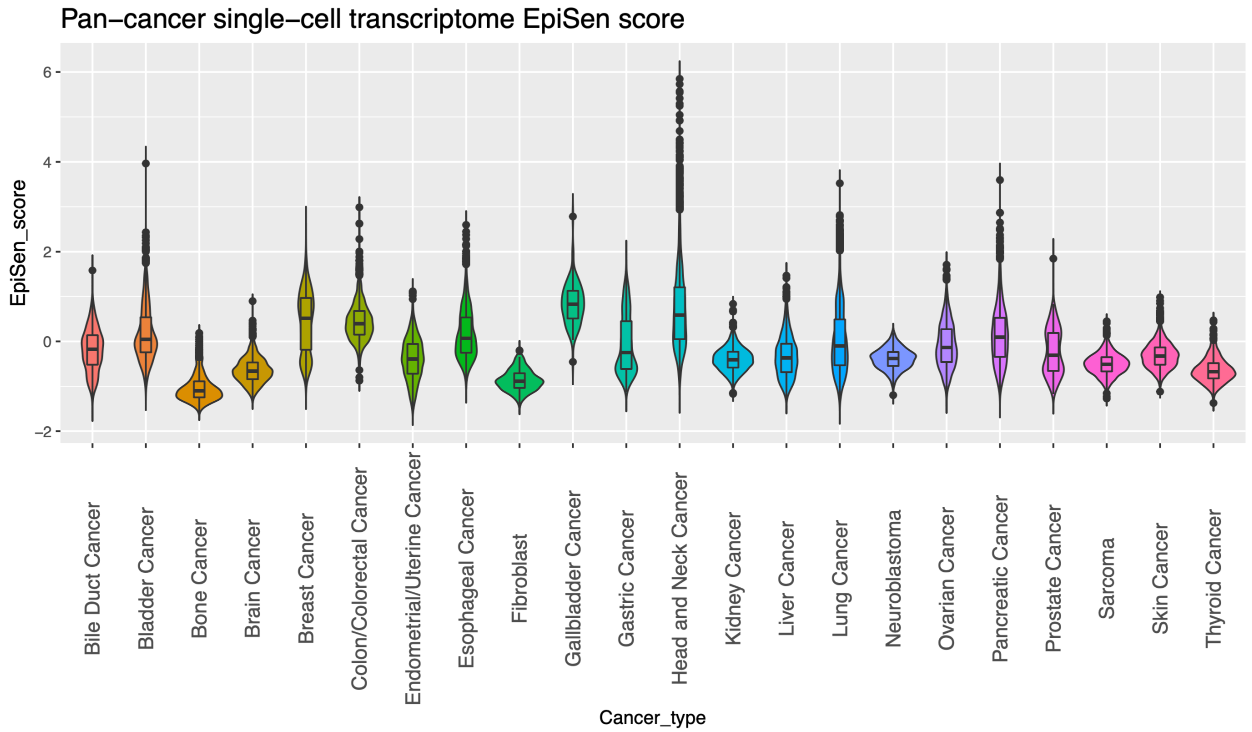

Fig. S4.
The violin plot of EpiSen Program score (doi:10.1038/s41588-020-00726-6) of single cells among twenty-two cancer types.

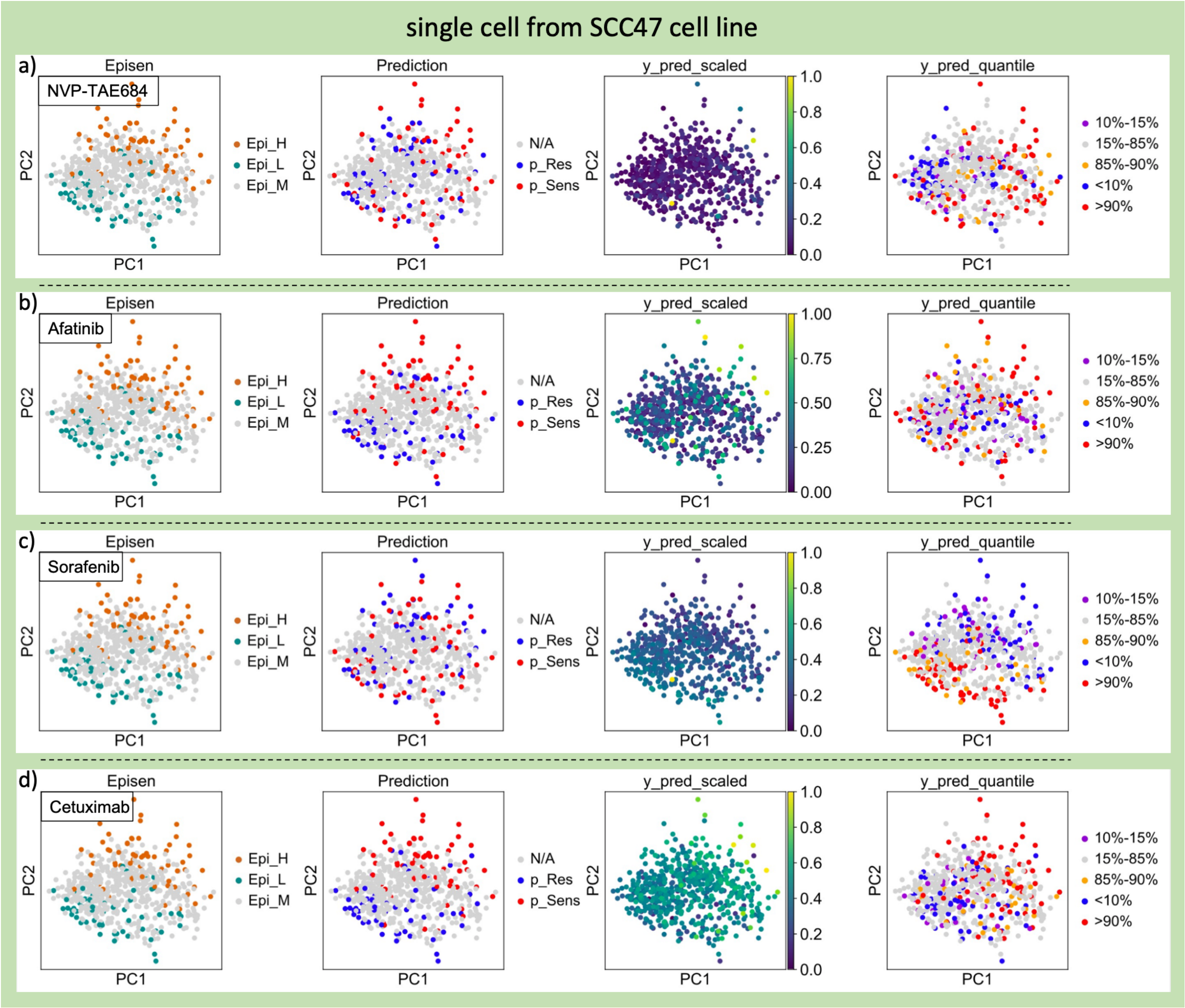

Fig. S5.

Drug resistance ranking of SCC47 cells. (a) The PCA dimensional reduction plot of single cells that colored by the EpiSen status. (b) The PCA plot of single cells that colored by the binarized (cutoff=median) SCAD prediction values. (c) Visualization of single cells by PCA projection which colored by the scaled SCAD prediction value after MinMaxScaler scale. (d) Visualization of single cells by PCA projection, which the SCAD prediction values of cells are stratified and colored by percentiles.

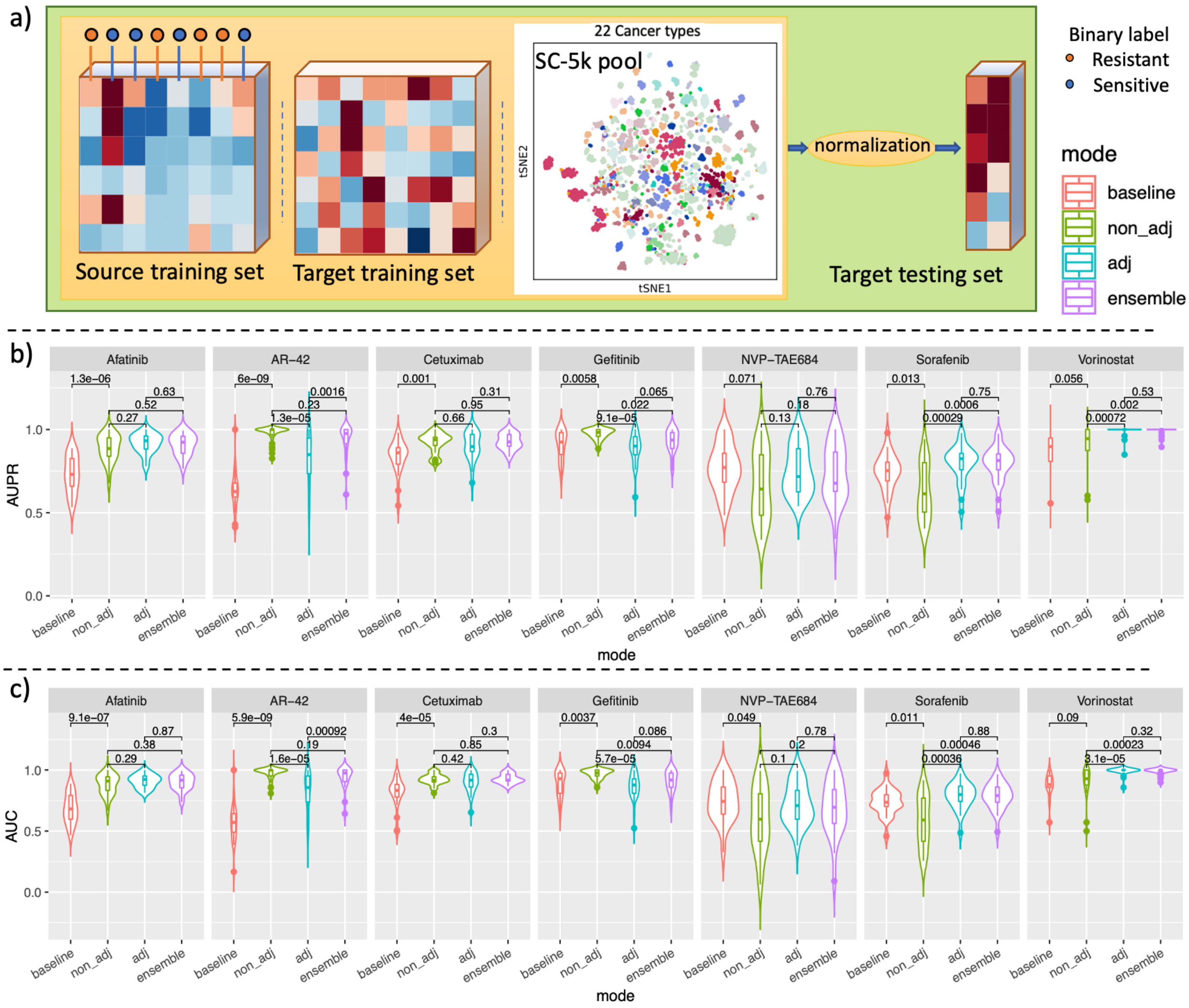

Fig. S6.

Normalizing source domain with SC-5K dataset. (a) The source domain training set and the target domain training set are normalized with SC-5K dataset (by sklearn.StandardScaler). Testing set are then transformed to get the adjusted data profile, the rest of data splitting are identical to the steps that described in Fig. 1a. After this data preprocessing step, the SCAD model was pre-trained following the architecture that presented in Fig. 1b. (b, c) Boxplot for AUC and AUPR comparisons of baseline, SCAD (non_adj), adj, ensemble models.

Supplementary method

Supplementary method for Fig. S6. A recent study suggested that pre-clinical cell lines (labelled) normalized with pre-clinical tumour samples (label unknown) could reduce the biological and statistical diversity between cell lines and tumour samples, elevating the drug efficacy prediction performance on the tumour samples of patients [1]. Inspired by this and to see if a similar strategy could enhance the prediction performance of SCAD, we normalized the bulk RNA-seq of GDSC

(source domain) with many independent scRNA-seq profiles in the data pre-processing step before model training. We randomly selected 10% of the SCP542 scRNA-seq profiles (after excluding cell line JHU006 and cell line SCC47 to avoid overlapping with the target domain by scanpy.pp.subsample() function [2] to obtain a single cell dataset (named as SC-5K) containing 5259 (5K) single cells (SC). As shown in Fig. S6a, the training set of the source domain and the training set of the target domain were combined and normalized with our randomly selected SC-5K dataset. The target testing set was then transformed with the above-normalized data distribution (Fig. S6a). The mean and standard deviation (SD) of the AUC and AUPR scores for each drug after SC-5K dataset normalization are summarized in Table S6 and Table S7. Following the same normalization and adjustment strategy, we found both average AUC and AUPR increases, suggesting that our findings complement and support the literature after following the reported strategy. Among all seven anti-cancer drugs, four of them (Vorinostat, NVP-TAE684, Afatinib, Sorafenib) obtained higher AUC and AUPR scores after normalizing with the SC 5K dataset (Table S6, Table S7). In addition, we observed elevated overall average AUC (0.855 vs. 0.831) and average AUPR scores (0.866 vs. 0.850) when evaluating all seven drugs after the normalization of the SC-5K dataset. One possible reason is that the SC-5K dataset normalization process could make the source domain predictor learn more informative features from the scRNA-seq to infer drug sensitivity for most compounds, since the hyper-parameters of SCAD are selected based on performance (5-folds AUC) of the validation set of the source domain (Table S5). We also compared the performance of baseline model (non-ADDA), non_adj (weight_all_ADDA), adj (weight_all_ADDA_adj), and ensemble (non_adj + adj). As we can see in Fig. S6b and Fig. S6c, only AR-42 and Gefitinib have decreased AUC and AUPR scores after SC-5K adjustment (non_adj vs. adj). Moreover, we also found that the variances of AUC and AUPR in ensemble mode are smaller than non_adj mode for most of drugs (e.g., Afatinib, NVP-TAE684, Sorafenib, and Vorinostat, Fig. S6b, Fig. S6c), suggesting that the performance of ensemble mode will be more stable than the counterparts.

[1] D. E. Hostallero, L. Wei, L. Wang, J. Cairns, A. Emad, bioRxiv 2021.
[2] F. A. Wolf, P. Angerer, F. J. Theis, Genome biology 2018, 19, 1 1.

Data S1. (Separate file)

Supplementary Table. S1 to Table. S7 in excel file.
